# Chronological age was not independently associated with ultra-early safety or arrhythmic outcomes after contemporary atrial fibrillation ablation: a real-world single-center study

**DOI:** 10.3389/fmed.2026.1831225

**Published:** 2026-07-07

**Authors:** Gennaro De Rosa, Marco Giuggia, Mattia Peyracchia, Martina Peddis, Roberto Di Summa, Elisa Pelissero, Giuseppe Trapani, Fabio Ugliano, Francesco Fiore, Giuseppe Corazzelli, Plinio Cirillo, Gaetano Senatore

**Affiliations:** 1 Division of Cardiology, Department of Advanced Biomedical Sciences, University of NaPles Federico II, Naples, Italy; 2 Ciriè Hospital, Ciriè, Italy; 3 Department of Advanced Biomedical Sciences, University of Naples Federico II, Naples, Italy; 4 Cardio-Oncology Unit, Department of Translational Medical Sciences, Center for Basic and Clinical Immunology Research (CISI), Interdepartmental Center of Clinical and Translational Sciences (CIRCET), Interdepartmental Hypertension Research Center (CIRIAPA), Federico II University, Naples, Italy; 5 Department of Neurosurgery, Presidio Ospedaliero “Santa Maria Delle Grazie”, Pozzuoli, Italy; 6 Department of Human Neurosciences, Sapienza University of Rome, Rome, Italy

**Keywords:** advanced age, arrhythmic recurrence, atrial fibrillation, catheter ablation, early outcomes, elderly patients, procedural safety

## Abstract

**Background:**

Transcatheter ablation (TCA) is an established treatment for atrial fibrillation (AF); however, the impact of age on procedural safety and ultra-early arrhythmic outcomes remains incompletely understood, particularly in real-world clinical settings.

**Objective:**

To assess the association between age and early procedural safety and ultra-early arrhythmic outcomes following AF ablation in an unselected real-world cohort.

**Methods:**

We retrospectively analyzed 217 consecutive patients (mean age 67 ± 9 years) who underwent transcatheter ablation for atrial fibrillation between January 2022 and December 2023 at a single tertiary center. Age was analyzed using three predefined cutoffs (< 65, < 70, and < 80 years) and as a continuous variable. Multivariable logistic regression analyses were performed to assess the independent association between age and procedural safety and ultra-early arrhythmic outcomes. The primary safety endpoint was a composite of pericardial effusion and/or cardiac tamponade confirmed by echocardiography, periprocedural bleeding requiring medical intervention or blood transfusion, stroke or transient ischemic attack (TIA), acute coronary events, cardiovascular or procedure-related death. The secondary arrhythmic endpoint was a composite of atrial fibrillation recurrence and AF persistence within 48 h. Both the composite endpoint and its individual components were evaluated in covariate-adjusted models.

**Results:**

No significant differences in early complications or arrhythmic outcomes were observed across the predefined age cutoffs. Multivariable logistic regression analysis showed that age was not independently associated with procedural safety (OR 0.99; 95% CI 0.93–1.06; *p* = 0.812), AF recurrence (OR 1.01; 95% CI 0.97–1.05; *p* = 0.77), or AF persistence (OR 1.00; 95% CI 0.96–1.05; *p* = 0.91).

**Conclusion:**

In this real-world, single-center cohort, age was not independently associated with procedural safety or ultra-early arrhythmic outcomes following AF ablation. These findings suggest that chronological age alone may not be a major determinant of ultra-early procedural outcomes in appropriately selected patients undergoing AF ablation.

## Introduction

Atrial fibrillation (AF) is the most common sustained cardiac arrhythmia worldwide and represents a major public health challenge. Contemporary estimates indicate that more than 59 million individuals are currently living with AF globally, with prevalence expected to increase substantially over the coming decades due to population ageing and improved survival from cardiovascular diseases. AF is associated with significant morbidity, mortality, reduced quality of life, and increased healthcare utilization (1). Its prevalence increases markedly with advancing age, reaching up to 10% among individuals older than 80 years (1). Transcatheter ablation (TCA) has become a cornerstone of rhythm-control strategies for patients with symptomatic AF who are refractory to or intolerant of antiarrhythmic drug therapy (1, 2). Compared with medical therapy alone, TCA has demonstrated superior efficacy in maintaining sinus rhythm, improving quality of life, and potentially reducing cardiovascular mortality and hospitalization (3, 4). Nevertheless, the impact of advanced age on the safety and long-term efficacy of AF ablation remains controversial (5). Elderly patients often present with a higher burden of comorbidities, increased periprocedural vulnerability, and more extensive atrial structural remodelling, factors that may theoretically increase complication rates and reduce procedural success (5). Conversely, progressive technological advances, refinement of ablation techniques, and improvements in periprocedural management have significantly reduced procedural risk, even in older populations (1, 6).

Several studies have investigated the relationship between age and patients’ clinical outcomes but results are still inconsistent. Some of them (5, 7) have shown a higher rate of complications and a lower arrhythmia-free survival in older patients (5, 7). On the contrary, other studies (8) and recent single-center experiences have not demonstrated significant age-related differences in procedural outcomes. These differences might be due to different study design, inclusion criteria, operator experience, and the evolving technological landscape over time. Given these inconsistencies, there remains a clear need for contemporary, homogeneous real-world data that reflect current clinical practice. Large multicenter registries often span extended time periods and involve heterogeneous protocols, potentially obscuring age-related differences in procedural outcomes. In this context, single-center analyses provide a complementary perspective by ensuring uniform procedural techniques, standardized periprocedural management, and consistent operator expertise (9).

The present study aimed to evaluate the association between age and early procedural safety and arrhythmic outcomes following AF ablation, with age analysed both as a continuous variable and across three clinically relevant thresholds (< 65, < 70, and < 80 years). This study provides contemporary real-world data from a single-center Italian cohort of 217 consecutive patients, reflecting homogeneous procedural strategies and standardized periprocedural management in current clinical practice. We hypothesized that age alone would not be independently associated with early (48-hour) procedural safety outcomes or arrhythmic recurrence. We further postulated that catheter ablation would remain a safe and effective therapeutic strategy across the age spectrum when performed in appropriately selected patients and experienced centers.

## Materials and methods

### Study population

This is a retrospective, single-center observational study conducted at the Cardiology Unit of Ciriè Hospital, Italy. The study was conducted and reported in accordance with the STROBE (Strengthening the Reporting of Observational Studies in Epidemiology) guidelines (10). The study population consisted of 217 consecutive patients who underwent transcatheter ablation for atrial fibrillation (AF) between January 2022 and December 2023. All procedures were performed by experienced electrophysiologists using contemporary ablation technologies and standardized periprocedural management protocols.

Patients were screened according to predefined inclusion and exclusion criteria.

**Inclusion criteria were:**

1. Age ≥ 18 years;
2. Diagnosis of paroxysmal or persistent AF according to the 2024 European Society of Cardiology (ESC) guidelines (1);
3. Patients undergoing first-time or repeat transcatheter ablation for rhythm control;
4. Availability of complete procedural and follow-up data.

**Exclusion criteria included:**

1. Presence of atrial flutter or other non-AF supraventricular arrhythmias as the primary ablation target;
2. Significant structural heart disease (e.g., severe valvular disease) or left atrial thrombus;
3. Active infection, decompensated heart failure, or pregnancy at the time of the procedure.

The study protocol complied with the Declaration of Helsinki (2013 revision). All patients provided written informed consent for the collection and anonymized analysis of clinical data. The procedural objective was pulmonary vein isolation in all patients. Additional lesion sets were performed only in selected cases according to operator discretion and underlying arrhythmia characteristics.

### Transcatheter ablation procedure

#### Procedural workflow

All patients were admitted to hospital at least 1 day before the expected procedure and underwent routine laboratory tests, a standard 12-lead ECG, a chest X-ray, Transesophageal Echocardiography (TEE) and pre-procedural anesthesiologic evaluation. Oral anticoagulation was managed according to contemporary guideline recommendations (1) and resumed within 4 h after the procedure in all patients. Pulmonary vein isolation (PVI) represented the cornerstone of the ablation strategy. PVI alone was performed in 174 patients (80.2%). Additional lesion sets were used in selected cases according to operator judgement and arrhythmia substrate characteristics, including cavotricuspid isthmus ablation in 35 patients (16.1%), roof line ablation in 4 patients (1.8%), posterior wall isolation in 1 patient (0.5%), combined posterior wall isolation and roof line ablation in 1 patient (0.5%), and combined mitral isthmus and roof line ablation in 2 patients (0.9%). Complex fractionated atrial electrogram (CFAE) ablation was not performed. Repeat ablation procedures accounted for 39 patients (18.0%), whereas 178 patients underwent a first ablation procedure. Ablation procedures were performed using contemporary radiofrequency (RF), cryoballoon, or laser technologies according to operator preference and clinical indication. Following transseptal access and systemic anticoagulation, electroanatomical mapping was performed when appropriate. Acute procedural success was defined as complete pulmonary vein isolation confirmed by standard electrophysiological criteria (11), including entrance and exit block. Acute pulmonary vein isolation was achieved in all patients.

#### Postprocedural care

Post-procedural transthoracic echocardiography was routinely performed to exclude pericardial effusion. Continuous in-hospital telemetric monitoring was performed for at least 48 h in all cases.

### Early (48-Hour) outcomes

Safety Outcomes

Early procedural safety outcomes were defined as any complication occurring within 48 h of the procedure, including:

- Pericardial effusion and/or cardiac tamponade confirmed by echocardiography;
- Periprocedural bleeding requiring medical intervention or blood transfusion;
- Stroke or transient ischemic attack (TIA);
- Acute coronary events;
- Cardiovascular or procedure-related death.

A composite safety endpoint was defined as the occurrence of any of these events within 48 h after the procedure.

Ultra-Early Arrhythmic Outcomes were assessed using two primary outcomes:

- AF recurrence, defined as any episode of AF or atrial tachyarrhythmia lasting ≥ 30 s, documented on ECG or continuous monitoring.
- AF persistence, defined as failure to restore or maintain sinus rhythm after transcatheter ablation and cardioversion, as documented on ECG monitoring.

### Statistical analysis

Statistical analyses were performed using IBM SPSS Statistics version 29 (IBM Corp., Armonk, NY, USA). Continuous variables were tested for normality using the Kolmogorov–Smirnov test and are presented as mean ± standard deviation (SD) or median with interquartile range (IQR), as appropriate. Categorical variables are expressed as absolute numbers and percentages.

Between-group comparisons were performed using the independent samples *t*-test or the Mann–Whitney U test for continuous variables and the χ^2^ test or Fisher’s exact test for categorical variables.

Patients were stratified according to three predefined age thresholds (< 65, < 70, and < 80 years), yielding dichotomous comparisons at each cut-off. In addition, age was analyzed as a continuous variable to assess potential linear associations with study outcomes.

Univariable and multivariable logistic regression models were constructed to assess the independent association between age and the primary and secondary endpoints. Given the limited number of outcome events, multivariable logistic regression models were constructed using a parsimonious approach to minimize the risk of overfitting. Adjustment was restricted to clinically relevant variables selected *a priori*, in accordance with events-per-variable recommendations.

Results are reported as odds ratios (ORs) with 95% confidence intervals (CIs). A two-tailed *p*-value < 0.05 was considered statistically significant.

Graphical analyses, including forest plots, were generated to visually explore the relationship between age and outcomes and to illustrate confidence intervals across predefined age groups.

## Results

### Baseline characteristics

A total of 217 consecutive patients were included in the analysis. The mean age was 67.2 ± 9.1 years (range, 42–84 years), and 141 patients (65%) were male. Paroxysmal AF was present in 133 patients (61.3%), whereas 84 patients (38.7%) had persistent AF.

The mean CHA_2_DS_2_-VASc score was 2.17 ± 1.6. Hypertension was reported in 125 patients (57.6%), and diabetes mellitus in 32 patients (14.7%).

Baseline characteristics stratified by a 65-year age cut-off are shown in Table 1. Patients aged ≥ 65 years exhibited a significantly higher prevalence of hypertension, diabetes mellitus, chronic kidney disease, and persistent atrial fibrillation compared with younger patients. Female sex was also more frequent in the older group. As expected, mean age differed significantly between groups (*p* < 0.001).

**TABLE 1:** Baseline clinical characteristics according to a 65-year age cut off.

Variable	< 65 (*n* = 117)	≥ 65 (*n* = 100)	*p*-value
Age, years (mean ± SD)	58.14 ± 5.1	72.8 ± 5.4	<0.001
Female sex, *n* (%)	32 (27.4%)	41 (41.0%)	0.032
Hypertension, *n* (%)	54 (46.2%)	71 (71.0%)	<0.001
Diabetes mellitus, *n* (%)	18 (15.4%)	26 (26.0%)	0.048
Chronic kidney disease, *n* (%)	9 (7.7%)	19 (19.0%)	0.014
Persistent AF, *n* (%)	37 (31.6%)	49 (49.0%)	0.009

Values are presented as mean ± standard deviation(SD) or number(percentage). Continuous variables were compared using student’s *t-*test. Categorical variables were compared using χ^2^ test or Fisher exact test when appropriate. AF, atrial fibrillation; CKD, chronic kidney disease; SD, standard deviation.

When stratified using a 70-year threshold, older patients continued to show a higher burden of hypertension, chronic kidney disease, and persistent atrial fibrillation. Differences in female sex and diabetes prevalence were attenuated and no longer statistically significant (Table 2).

**TABLE 2:** Baseline clinical characteristics according to a 70-year age cut-off.

Variable	<70 (*n* = 143)	≥ 70 (*n* = 74)	*p*-value
Age, years (mean ± SD)	61.2 ± 6.3	74.9 ±	<0.001
Female sex, *n* (%)	41 (29.1%)	29 (39.0%)	0.11
Hypertension, *n* (%)	71 (49.6%)	50 (67.5%)	0.008
Diabetes mellitus, *n* (%)	23 (16.2%)	18 (24.3%)	0.09
Chronic kidney disease, *n* (%)	12 (8.5%)	14 (18.9%)	0.034
Persistent AF, *n* (%)	48 (33.3%)	35 (47.2%)	0.038

Values are presented as mean ± SD or number (percentage). Continuous variable were compared using student’s *t-*test categorical variables were compared using *x*^2^ test or Fishers exact test when appropriate. AF, atrial fibrillation; CKD, chronic kidney disease; SD, standard deviation.

Only seven patients were aged ≥ 80 years. In this subgroup, hypertension was universally present. Other comorbidities appeared numerically higher but did not reach statistical significance, likely due to limited statistical power (Table 3).

**TABLE 3:** Baseline clinical characteristic According to an 80-year age cut off.

Variable	< 80 (*n* = 210)	≥ 80 (*n* = 7)	*P*-value
Age, years (mean ± SD)	65.2 ± 8.7	82.4 ± 2.1	<0.001
Female sex, *n* (%)	71 (33.8%)	2 (28.6%)	0.78
Hypertension, *n* (%)	11 (56.2%)	7 (100%)	0.018*
Diabetes mellitus, *n* (%)	42 (20.0%)	2 (28.6%)	0.62
Chronic kidney disease, *n* (%)	26 (12.4%)	2 (28.6)	0.24
Persistent AF, *n* (%)	81 (38.6%)	5 (71.4%)	0.10

Values are presented as mean ± SD or number (percentage). Continuous variables were compared using student’s *t-*test. Due to small sample size in the ≥ 80 years group (*n* = 7), Fisher’s exact test was used where appropriate. AF, atrial fibrillation; CKD, chronic kidney disease; SD, standard deviation. *Indicates statistical significance (*p* < 0.05).

### Procedural characteristics and early safety outcomes

All transcatheter ablation procedures were successfully completed, and acute pulmonary vein isolation was achieved in 100% of patients.

Early procedural safety was favorable, with a low overall rate of complications within 48 h after the procedure. Specifically, pericardial effusion and/or cardiac tamponade occurred in 13 patients (6.0%), and periprocedural bleeding in 6 patients (2.8%). No procedure-related deaths, stroke, transient ischemic attack, or acute coronary events were observed during the early post-procedural period.

When safety outcomes were stratified according to predefined age categories, no statistically significant differences were observed across any cut-off. The incidence of the 48-hour composite safety endpoint was comparable between patients aged < 65 versus ≥ 65 years (*p* = 0.68), < 70 versus ≥ 70 years (*p* = 0.74), and < 80 versus ≥ 80 years (*p* = 0.83).

Analysis of individual safety components similarly showed no significant age-related differences. Bleeding events were infrequent and evenly distributed across age strata. BARC 2 bleeding at 48 h was not associated with age (χ^2^ = 1.528; *p* = 0.216), and BARC 3a bleeding remained rare across all age groups (χ^2^ = 0.399; *p* = 0.528). Ischemic stroke was not observed within 48 h, precluding comparative analysis. Vascular complications and pericardial interventions occurred sporadically and without significant variation across age groups.

**FIGURE 1:**
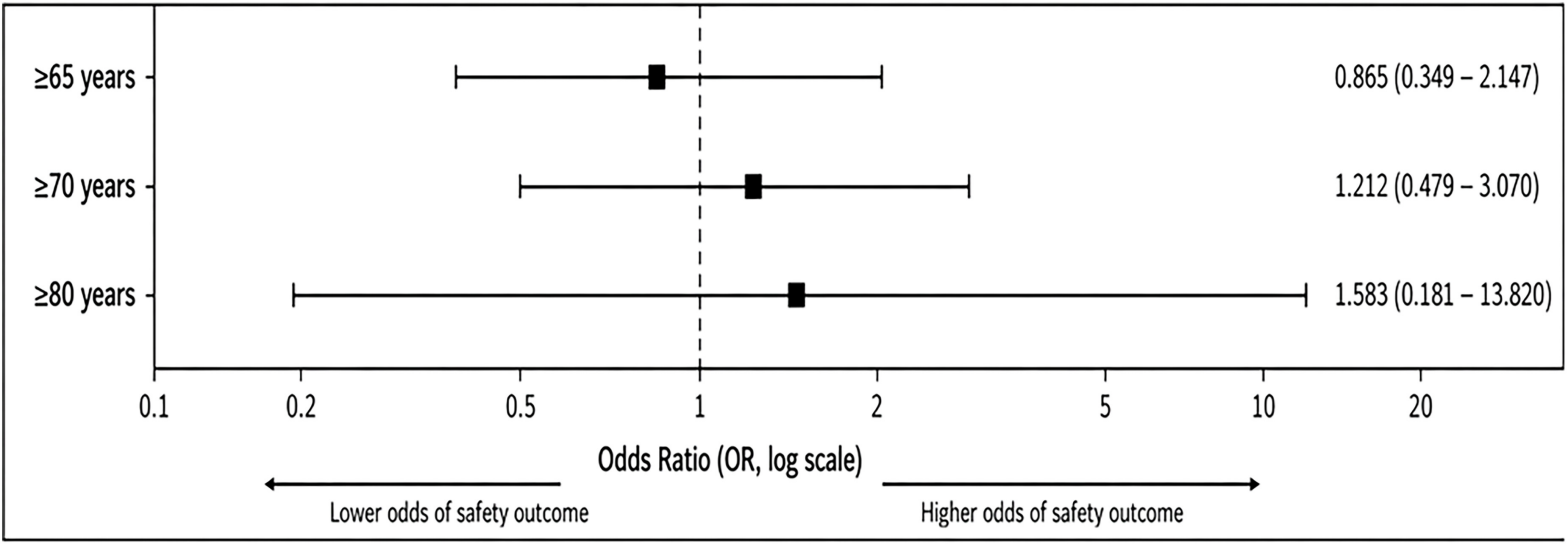
Association between age thresholds and 48 h Safety outcomes following AF ablation. Forest plot displaying odds ratios (ORs) and 95% confidence intervals (CIs) for the composite 48-hour safety end point according to predefined age thresholds. The dashed vertical line represents the null value (OR=1).

In a parsimonious multivariable logistic regression model adjusted for CHA_2_DS_2_-VASc score, chronological age was not independently associated with 48-hour safety outcomes (OR 0.99 per 1-year increase, 95% CI 0.93–1.06; *p* = 0.812) (Table 4). To address potential concerns regarding overlap between age and the CHA_2_DS_2_-VASc score, an additional sensitivity analysis was performed using female sex and persistent AF as alternative covariates. The results remained materially unchanged, with no significant association observed between age and the composite safety endpoint (OR 0.98 per 1-year increase, 95% CI 0.934–1.028; *p* = 0.402). Forest plot analysis across the predefined age thresholds (≥ 65, ≥ 70, and ≥ 80 years) demonstrated no significant association between advancing age and the composite 48-hour safety endpoint. Odds ratios remained close to unity across all thresholds, with progressively wider confidence intervals observed at higher age cut-offs, reflecting the limited number of patients in the oldest subgroup (Figure 1).

**TABLE 4:** Multivariable logistic regression analysis for the 48-hours composite safety end point.

Predictor	OR	95%CI	*p*-value
Age (per1-year increase)	0.99	0.93–1.06	0.812
CHA_2_DS_2_-VASc score	0.96	0.65–1.42	0.826

Values are presented as odd ratios (ORS) with 95% confidence intervals (CIS). The models was adjusted for clinically relevant covariates. Calibration was adequate (Hosmer–Lemeshow *p* = 0.919) No multicollinearity was detected (VIF < 2).

### Ultra-early arrhythmic outcomes

The ultra-early arrhythmic events evaluated in the present study should not be interpreted as direct measures of long-term procedural efficacy, as arrhythmias occurring within the first 48 h after ablation are generally considered part of the blanking period and may reflect transient procedural effects rather than durable lesion failure.

At 48 h following ablation, AF recurrence occurred in 23 patients (10.5%), while AF persistence was observed in 8 patients (3.7%).

When stratified by age categories, recurrence and persistence rates were similar across all predefined thresholds. AF recurrence did not differ significantly between patients aged < 65 versus ≥ 65 years (*p* = 0.59), < 70 versus ≥ 70 years (*p* = 0.68), or < 80 versus ≥ 80 years (*p* = 0.77). Likewise, AF persistence showed no significant variation across age groups (*p* = 0.73, *p* = 0.66, and *p* = 0.81 for the < 65, < 70, and < 80 cut-offs, respectively).

**FIGURE 2:**
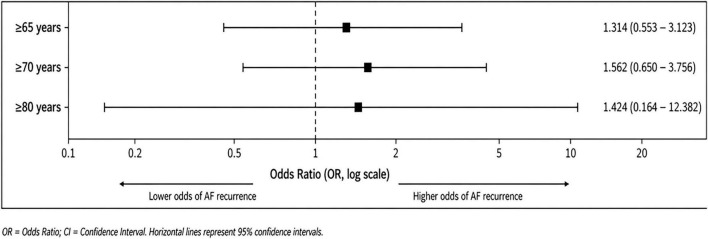
Association between age threshold and ultra-early AF recurrence following AF ablation. Forest plot displaying odds ratios (ORs) and 95% confidence intervals (CIs) for AF recurrence within 48 h according to predefined age threshold. The dashed vertical line represent the null value (OR = 1).

In multivariable logistic regression analyses treating age as a continuous variable, no independent association was observed between age and early arrhythmic outcomes. Forest plot analysis across predefined age thresholds demonstrated no significant association between age category and ultra-early AF recurrence (Figure 2). Consistent with these findings, a parsimonious model including CHA_2_DS_2_-VASc score was applied, in accordance with the primary analysis. The odds ratio per year increase in age was 1.01 (95% CI 0.97–1.05; *p* = 0.77) for AF recurrence and 1.00 (95% CI 0.96–1.05; *p* = 0.91) for AF persistence.

## Discussion

### Principal findings

In this contemporary real-world cohort, age was not independently associated with procedural safety or ultra-early arrhythmic outcomes following AF ablation. These findings remained consistent when age was analyzed both categorically (< 65, < 70, and < 80 years) and as a continuous variable and persisted after multivariable adjustment using a parsimonious model including CHA_2_DS_2_-VASc score. Overall event rates were low, with no periprocedural deaths, no cerebrovascular events, and an overall complication rate of 6.9%. Importantly, no significant age-related gradient in procedural risk was observed across any predefined threshold.

Taken together, these results suggest that chronological age alone may not represent a determinant of early procedural risk or short-term arrhythmic outcomes in contemporary AF ablation practice when patients are carefully selected and procedures are performed in experienced centers.

Importantly, ultra-early outcomes within 48 h may serve as indicators of procedural robustness and the quality of periprocedural care pathways. Ultra-early outcomes primarily reflect procedural integrity and periprocedural management rather than long-term atrial substrate characteristics. The absence of major early adverse events among older patients in our study supports the notion that contemporary ablation workflows can achieve consistent safety profiles across a broad age spectrum.

### Comparison with previous studies

#### Safety in elderly populations

Historically, advanced age was considered a relative contraindication to AF ablation, largely based on earlier data suggesting higher complication rates among older adults (7). However, contemporary large-scale studies have demonstrated favorable safety profiles even in elderly populations (5).

The CABANA trial reported a 30-day major complication rate of 1.7% and did not identify significant age-related differences in procedural safety (12). Registry analyses have consistently identified center volume and operator experience as major determinants of periprocedural safety in AF ablation (9, 13). In the elderly subgroup analysis of the FIRE AND ICE trial, complication rates among patients aged ≥ 75 years were comparable to those observed in younger patients (4.3% vs 3.8%) (8).

Our findings are consistent with these observations. In our cohort, pericardial effusion occurred in 6.0% of patients, and vascular complications in less than 2%. No stroke or procedure-related death was observed. These data suggest that, under contemporary procedural standards, age *per se* may not be associated with an increased short-term procedural risk.

Collectively, these findings suggest that procedural safety may be more closely associated with operator expertise, technological advancements, and standardized periprocedural management than with chronological age alone.

### Early arrhythmic outcomes

Our data did not reveal significant differences in early arrhythmic recurrence or persistence across age strata. Early AF recurrence within 48 h was observed in 10.5% of patients, consistent with the expected early “blanking period” phenomenon described in prior studies (11, 14).

Previous investigations have suggested that immediate post-procedural AF recurrences often reflect transient inflammation, autonomic imbalance, or atrial stunning rather than durable pulmonary vein reconnection, and may therefore not predict long-term rhythm outcomes (15, 16).

Although advancing age has been associated with more extensive atrial structural remodeling and fibrosis (17, 18), these chronic substrate-related factors may exert a lesser influence during the acute post-ablation phase, when arrhythmogenesis is predominantly driven by procedural variables such as contact force optimization, lesion durability, and transient atrial stunning (11, 19).

The absence of significant age-related differences in our cohort—including among patients aged ≥ 80 years—suggests that early procedural efficacy and short-term rhythm stabilization are unlikely to be substantially influenced by chronological age (5).

### Mechanistic and physiological context

Several mechanisms may explain the absence of an age-related increase in short-term procedural risk observed in our cohort. First, contemporary technological advancements—including contact-force sensing catheters, high-power short-duration (HPSD) radiofrequency strategies, and real-time oesophageal temperature monitoring—have enhanced lesion reproducibility and improved overall procedural safety (1, 19). Second, periprocedural anticoagulation management has become increasingly standardized; uninterrupted direct oral anticoagulant (DOAC) strategies have been associated with a reduction in both thromboembolic and bleeding complications (20, 21). Third, operator experience and institutional procedural volume are recognized determinants of safety in atrial fibrillation ablation, with high-volume centers consistently reporting lower complication rates regardless of patient age (9, 13).

Collectively, these factors may mitigate the potential impact of age-related physiological vulnerability during the periprocedural phase.

### Clinical implications

Our findings have relevant implications for clinical decision-making and patient counselling. As the prevalence of atrial fibrillation continues to increase with population aging, the number of elderly patients considered for catheter ablation is expected to rise accordingly (1). In this scenario, the traditional perception of advanced age as a relative procedural contraindication warrants critical reappraisal considering contemporary evidence (5).

Accumulating data—including the present analysis—suggest that procedural candidacy should be guided more appropriately by biological age, frailty status, and overall comorbidity burden rather than chronological age *per se* (22). In our cohort, even among octogenarians, advanced age was not associated with a higher risk of early complications, nor did it necessitate modifications in standard post-procedural management strategies (5, 8).

Recent evidence has further suggested that the timing of catheter ablation may represent a more important determinant of procedural and long-term rhythm outcomes than chronological age itself (23). In particular, longer diagnosis-to-ablation intervals have been associated with less favorable outcomes, potentially reflecting progressive atrial remodelling and increasing arrhythmia burden over time (23). From this perspective, delaying referral solely on the basis of advanced age may be inappropriate and could inadvertently reduce the likelihood of achieving optimal outcomes. Therefore, contemporary decision-making should consider not only patient age, but also disease duration, atrial substrate characteristics, frailty status, and overall clinical profile when evaluating candidacy for catheter ablation (1, 22, 23).

Another clinically relevant consideration in patients undergoing AF ablation, particularly older individuals, is the long-term management of oral anticoagulation. Although the present study was not designed to evaluate post-ablation anticoagulation strategies, successful rhythm control may influence decisions regarding long-term anticoagulant therapy in selected patients. Nevertheless, contemporary evidence suggests that anticoagulation decisions should remain individualized and primarily guided by thromboembolic risk assessment rather than rhythm outcome alone (1, 24). Consequently, maintenance or discontinuation of oral anticoagulation after ablation should be based on an integrated evaluation of patient-specific risk factors and not solely on apparent procedural success or short-term rhythm status (1, 24).

These findings align with the evolving paradigm of personalized atrial fibrillation management, in which therapeutic decisions are increasingly tailored according to symptom burden, AF phenotype, atrial substrate characteristics, and comorbidity profile rather than chronological age alone (1, 22, 23).

### Limitations and future directions

Several limitations of the present study should be acknowledged.

First, the retrospective single-center design may limit the generalizability of our findings to broader and more heterogeneous populations. Nonetheless, the use of homogeneous patient selection criteria and standardized procedural workflows may enhance internal validity and reduce variability related to operator technique and periprocedural management.

Second, although the overall low event rate reflects contemporary procedural safety standards, it inevitably reduces statistical power and limits the ability to detect small but potentially clinically meaningful differences across age strata. Owing to the relatively low number of events, multivariable analyses should be interpreted as exploratory and hypothesis-generating rather than definitive.

Third, the analysis was confined to ultra-early (48-hour) outcomes. While ultra-early post-procedural events are clinically relevant and may reflect procedural quality, delayed complications and long-term arrhythmic recurrences were not evaluated. Consequently, no conclusions can be drawn regarding mid- or long-term age-related differences in efficacy or safety.

Furthermore, the limited number of outcome events constrained the complexity of multivariable adjustment. Consequently, parsimonious regression models were adopted to reduce the risk of overfitting. Although a sensitivity analysis using alternative covariates yielded consistent results, the findings should be interpreted as exploratory and hypothesis-generating.

Important modifiers of procedural risk—such as validated frailty indices, comprehensive left atrial structural parameters (e.g., indexed left atrial volume or fibrosis burden), and detailed procedural metrics including total ablation time and fluoroscopy exposure—were not systematically incorporated. Integration of these variables into future prospective studies could further refine risk stratification, particularly in elderly populations.

Another limitation of the present study is the absence of patient-reported outcome measures, including quality of life, symptom burden, functional status, and perceived well-being. These factors are particularly relevant in elderly patients, in whom the decision to undergo catheter ablation is often driven not only by rhythm control objectives but also by the potential for symptom relief, preservation of autonomy, and improvement in daily functioning. Future investigations should incorporate validated quality-of-life and symptom assessment instruments to provide a more comprehensive evaluation of the clinical benefits of AF ablation across different age groups (25).

Notably, confidence intervals widened considerably at the ≥ 80-year threshold for both safety and ultra-early arrhythmic outcomes, highlighting the limited precision of estimates in octogenarians and reinforcing the need for cautious interpretation of subgroup analyses in this population.

Future multicenter prospective registries should incorporate standardized frailty assessments, biological age markers, and extended longitudinal follow-up beyond the immediate post-procedural phase. In addition, the establishment of national or European datasets systematically capturing ultra-early (≤ 48-hour) outcomes may help clarify the independent contribution of age to procedural safety and inform evidence-based discharge strategies in older patients.

## Conclusion

In this contemporary real-world cohort, chronological age was not independently associated with ultra-early (48-hour) procedural safety or arrhythmic outcomes following atrial fibrillation ablation. These findings were consistent across multiple predefined age thresholds and persisted after adjustment using a parsimonious model including CHA_2_DS_2_-VASc score.

Collectively, our results suggest that no statistically significant association between chronological age and ultra-early procedural safety or arrhythmic outcomes was observed in this cohort. However, given the limited number of outcome events and the small number of octogenarians, larger studies are required to exclude modest age-related differences with greater confidence. Prospective multicenter studies involving larger and more heterogeneous populations are warranted to validate these observations and to further explore the integration of biological age, frailty indices, and structural cardiac parameters into individualized risk stratification frameworks for AF ablation.

## Data Availability

The raw data supporting the conclusions of this article will be made available by the authors, without undue reservation.
